# An analytical solution to ecosystem-based F_MSY_ using trophic transfer efficiency of prey consumption to predator biological production

**DOI:** 10.1371/journal.pone.0276370

**Published:** 2022-11-10

**Authors:** Bruce R. Hodgson

**Affiliations:** School of Environment, Science and Engineering, Southern Cross University, Lismore, New South Wales, Australia; Swedish University of Agricultural Sciences and Swedish Institute for the Marine Environment, University of Gothenburg, SWEDEN

## Abstract

A theoretical basis for Ecosystem-based Fisheries Management (EBFM) was derived for pelagic fish by applying marine ecology theory of analytical relationships of predator-prey biological production transfers between trophic levels to FAO guidelines for an ecosystem approach to fisheries. The aim is to describe a simple method for data-limited fisheries to estimate ecosystem-based F_MSY_ and how EBFM modellers could mimic the way natural fish communities function for maintaining ecological processes of biological production, biomass and ecosystem stability. Ecosystem stability (ES) F_MSY_ were estimated by proportion of biological production allocated to predators, giving ^ES^F_MSY_ of 0.23 for small pelagic and 0.27 for pelagic finfish, prioritising ecosystem over economics. To maintain both stability and biomass (SB) a full pelagic EBFM ^SB^F_MSY_ of about 0.08 was obtained for both small pelagic and pelagic finfish, having mostly ecosystem considerations. As the F_MSY_ are single-species averages of catchable species targeted in a specific trophic level, multispecies fishing mortalities were proportioned by the biological production of each species in the trophic level. This way catches for each species are consistent with the average ecosystem F_MSY_ for a trophic level. The theoretical estimates gave similar results to other fisheries for sustainable fish catches that maintain the fishery ecosystem processes. They were also tested using six tropical Ecopath Models and showed the effects of imposing commercial fishing mortalities on predominantly EBFM conditions. The ecosystem stability ^ES^F_MSY_ is suggested to be investigated for sustainable fish catches and the full EBFM ^SB^F_MSY_ for protected areas or recovery of heavily depleted stocks.

## Introduction

Although considerable progress has been made in development of Ecosystem-Based Fisheries Management (EBFM), its implementation has been slow [1], apparently due to the complexity of including many ecosystem and socio-economic factors [2, 3]. The review by [4] indicates that progress has been made using ecosystem models and developing corresponding reference points and indicators as part of an ecosystem-based harvest strategy. However, there has been a problem with limited application of ecosystem modelling to EBFM, apparently because the fundamentals of how they work are not well understood [5]. Hence, the goal of this paper is examines how natural fish community ecosystems function according to marine ecology theory [6] of biological production transfers between trophic levels (TL). The theory has biological production transferred between TLs by the Trophic Transfer Efficiency (TTE), typically _~_10%, the ratio of predator biological production to prey biological production. This way, prey biological production is transferred to the predator production by consumption of the prey production [7]. The aim is show how this works by fisheries mimicking the way natural fish communities function for maintaining ecological processes of biological production, biomass and ecosystem stability. Those processes are resolved by deriving an analytical relationship of predator-prey biological production transfers between trophic levels. Hence, modelling for EBFM could include those ecological processes for biomass and ecosystem stability. In this regard, the relative stability of unfished populations has been confirmed by [8] as an important factor to consider when assessing the effects of fishing. Hence, ecosystem stability has been investigated here using marine ecology theory. These processes are the fundamental basis of how a natural fish ecosystem operates to maintain a stable fish community and is proposed to represent the basis of an ecosystem-based fishery.

Therefore, pelagic ecosystem-based fisheries could mimic natural fish community processes and thereby help define how fishing mortalities could be estimated and applied to EBFM. The use of trophic transfer efficiency of prey biological consumption to predator biological production as an uncomplicated means of estimating ecosystem-based fishing mortalities was suggested by [9]. They proposed defining the level of fishing that yields the maximum productivity of the fish stock to human consumers while supporting the natural predators. This was followed by [10], who suggested the ecosystem component of EBFM may be resolved by applying marine ecological studies to uncomplicated ecosystems, such as those defined by trophic levels or other groups. The trophic level approach has been used here to estimate ecosystem-based fishing mortalities for small pelagic and pelagic finfish. To minimise complexity, the ecosystem processes of biological production transfers are applied to pelagic fisheries. Transfers begin with zooplankton production (in TL2) to the small pelagic, SP, production in TL3. This is followed by transfer of SP production to pelagic finfish, PF, predators (in TL4), and then PF production to the biological production of top predators in TL5 (mostly sharks). Note that the biological production of each fished species, groups or taxa, in a TL is estimated by the basic Ecopath Model using the fishery biomass multiplied by the estimated P/B ratio [11]. This approach for estimating EBFM conditions by using predator-prey biological production transfers between trophic levels is examined here for consistency with the FAO guidelines for an ecosystem approach to fisheries outlined by [12].

### The ecosystem stability ^ES^F_MSY_ and full EBFM ^SB^F_MSY_ concepts

The F_MSY_ concept is used here to describe how to apply ecosystem-based conditions to F_MSY_. This is undertaken by using the F_MSY_, with spawning stock recruitment (SSR), ^SSR^F_MSY_, to establish a starting point for application of ecosystem conditions to EBFM fishing mortalities. F_MSY_ is widely used for fishery management, with adjustments for changes in the spawning stock biomass (SSB) to maintain recruitment, and estimation of EBFM fishing mortalities [13]. By allowing for recruitment, the F_MSY_ was shown by [14] to be compatible with EBFM conditions. They also presented some confirmation that the lower fishing mortality could give an increased fishery biomass able to maintain ecological stability of prey and predators. Practical applications of applying ecosystem conditions to F_MSY_ by allowing for recruitment by the SSB, biological interactions as well as the effects of fishing on predators and prey species is used in the Greater North Sea Eco region by [15]. Furthermore, F_MSY_ is based on the assumption of steady state conditions where the harvest is in equilibrium with increased population growth rate [16]. Steady state conditions also occur in natural fish communities because the predators consume the biological production of their prey [7], not the underlying biomass that generates prey production. These ecosystem conditions mean the observed natural biomass variability of a fishery is mostly due to changes in SSB and environmental conditions in the fishery area [17]. Furthermore, steady state conditions mean that the total mortality, Z, equals the biological production to biomass ratio (P/B), the rate of biomass regeneration [18], which is used in Ecopath Models [11, 19]. These processes are consistent with the observations by [20], their Appendix 1, Section 1, that biological production is fundamental to fishery management because the fish catch is a proportion of the average biomass, as well as a proportion of the biological production. This was confirmed by [21] that stock production is the main ecosystem driver for fish catch.

Accordingly, the EBFM fishing mortalities are estimated here assuming the catch is directly related to the fishery biological production, under steady state and ecosystem-based conditions that mimic natural fish community processes. The F_MSY_ values are estimated by having an upper limit to F_MSY_ of 0.5 and having some of the fish catch forgone in proportion to the biological production of the fishery required to be transferred to predators to maintain ecosystem stability and the fishery biomass. The assumption is considered reasonable because the fish catch, and hence F_MSY_, is directly related to biological production.

The analytical relationship of predator-prey biological production transfers allows estimation of EBFM fishing mortalities by reducing the spawning stock recruitment ^SSR^F_MSY_ mortalities by the factor $\sqrt{TTE}$. The resulting fishing mortalities for ecosystem stability, ^ES^F_MSY_, and for full EBFM with stability and biomass, ^SB^F_MSY_, are shown how to be calculated by examples in the Methods Section and its application to twelve tropical Ecopath Model fisheries and nine temperate fisheries in the Results Section. As the F_MSY_ is a single-species fishing mortality, the fishing mortalities are averages of the catchable fish in a specific trophic level. To provide for a multispecies fishery, inside a trophic level, the average fishing mortality is proportioned for each species with the estimated EBFM F_MSY_ values. An example of estimating fishing mortalities for a trophic level based multispecies fishery is shown in the Methods and is considered simple enough to be used for data-limited fisheries.

## Methods

To determine the Ecosystem-Based F_MSY_ values for pelagic marine fisheries of small pelagic fish and their pelagic finfish predators, the analyses undertaken are: (i) determine the trophic transfer efficiencies from prey to predator from their estimated or typical trophic levels, (ii) to support predators, estimate the proportion of prey biological production transferred to support production of predators, (iii) allocate to the fishery the proportion of production not diverted to support predator production, (iv) estimate the ecosystem stability ^ES^F_MSY_ by the proportion of production allocated to the fishery under the conditions of not exceeding 0.5 and applying precautionary factors to allow for spawning stock recruitment, (v) estimate the ecosystem and biomass stability ^SB^F_MSY_ by further reducing the proportion of production allocated to the fishery by foregoing the prey production supporting production of the species being fished. In that way, the fishery not only supports the predators but only consumes the prey production, rather than the biomass generating the production. These ecological processes of biomass and ecosystem stability applies to the trophic transfer efficiencies of marine fisheries that tend to prey on and are preyed upon by pelagic fish, and in the case of TL3 species (small pelagic fish, also called forage fish) that mainly prey on zooplankton.

In order to estimate EBFM F_MSY_ values by marine ecology theory, the following relationships and conditions are defined: (i) analytical relationship of predator-prey biological production transfers between trophic levels, (ii) relationship between TTE and TL, (iii) F_MSY_ upper limit of 0.5 used as a baseline for estimation of ^SSR^F_MSY_ values and EBFM F_MSY_ for ecosystem stability and maintenance of the fishery biomass, (iv) established precautionary factors, PF, used to estimate ^SSR^F_MSY_ for spawning stock recruitment by application to the 0.5 baseline, (v) proportioning F_MSY_ values between multispecies fished in a trophic level. The basis of ecosystem-based fishing mortality estimations are included to describe how the above ^ES^F_MSY_ concepts apply to EBFM. The assumptions used and examples for estimating ecosystem-based ^ES^F_MSY_ and ^SB^F_MSY_ are also included in the Methods.

As items (i) to (iv) are known or can be estimated, a simple method is proposed to estimate ecosystem-based F_MSY_ values for data-limited fisheries. Item (v), estimation of ecosystem-based multispecies fishing mortalities is not suitable for data-limited fisheries because the multispecies procedure requires knowing the existing biomass and fishing mortalities of each species fished in a trophic level. A proposed definition of ecosystem stability is also provided in the Methods because it relates to estimation of EBFM F_MSY_ for allocation of biological production to support both the prey and predators in a fishery.

### Predator-prey biological production transfers

To estimate what reductions in the F_MSY_ are required to allow for predator consumption of their prey, it is necessary to know how to estimate prey consumption between tropic levels. Essentially, it is necessary to know the amount of prey biological production consumed by predators that goes to the predator’s biological production. The theoretical study on ecosystem functioning by [18] shows a conceptual model where the consumption transfer of prey biological production to the predator trophic level is reduced by the effects of excretion and respiration, the residual food transfer giving the predator biological production. This implies the amount of a predator’s consumption of their prey’s biological production is related to the prey’s biological production. For example, the TTE, between trophic levels, for prey production (Pprey) to predator production (Ppred) is given by the original definition in [7] with its application to fish and fisheries by [28] in Section “Relationship of trophic transfer efficiencies with trophic levels” below, giving Eq 1:

$$TTE=\frac{Ppred}{Pprey}$$ (1)

To put the trophic transfer efficiency into the context of predator consumption, Qpred, of the prey biological production, Qpred, is included in Eq 1 by:

$$TTE=\frac{Qpred}{Pprey}x\frac{Ppred}{Qpred}$$ (2)

Note that Qpred is not the total consumption by predators but the amount of prey biological production consumed that goes to predator biological production in the next higher TL. In words, the first ratio is the predator consumption of prey production, Qpred, relative to the prey production, Pprey. The second ratio is the resulting predator production, Ppred, relative to the prey consumption, Qpred. Substituting Ppred = TTE x Pprey and Prey = Pred/TTE from Eq 1 into Eq 2 gives:

$$TTE=\frac{QpredxTTE}{Ppred}x\frac{TTExPprey}{Qpred}$$ (3)

The common numerator TTE in the substitutions means both parts of Eq 2 are related to the square root of TTE because TTE^1/2^ x TTE^1/2^ = TTE. Therefore, from the first part of Eq 2, $\frac{Qpred}{Pprey}=\sqrt{TTE}$, so Qpred is related to Pprey by:

$$Qpred=Pprey\;x\;\sqrt{TTE}$$ (4)

Similarly, the second part of Eq 2 is $\frac{Ppred}{Qpred}=\sqrt{TTE}$. This means the predator production, Ppred, is related to the prey consumption, Qpred, by:

$$Ppred=Qpred\;x\;\sqrt{TTE}$$ (5)

Note that substituting Qpred from Eq 4 into Eq 5 gives Ppred = Prey x TTE, the same as the basic Eq 1 from marine ecology principles for a typical 10% trophic transfer efficiency. Those relationships show prey production consumption by Eq 4, over a nominal period of one year, feeds into predator production via Eq 5, giving the calculation of TTE by Eq 1 and the analytical equations allow estimation of the predator-prey biological production transfers between trophic levels. Please note the consumption of prey production by predators (t/Km^2^/year) by Eq 4 is not the same as the consumption of individual prey by predators according to [22] nonlinear, saturating effects of predator consumption of individual prey densities. The linear relationship of consumption of prey production with predator production by Eqs 4 and 5 are shown in the Section on “Ecopath model fishery examples to test EBFM theory”, below. Hence, the factor $\sqrt{TTE}$ is proposed as the key to understanding how the natural fish community ecosystems function and its application for estimation of ecosystem-based fishing mortalities.

#### Basis of the ecosystem-based fishing mortality estimations

The following provides the basis for estimation of the ecosystem-based fishing mortalities. As an example, the typical TTE of 0.10 (10%) gives $\sqrt{TTE}$ = 0.316, about 30% prey biological production transfer to support predator biological production, which is similar to that measured by [23], their page 337. They found about 29±6% of the food consumed by fish goes to growth and maintenance of respiration and excretion, with most of the food energy used for maintenance. Using the recent Ecopath and Ecosim (EwE) model, [24] showed the respiration/food assimilation ratios (assimilation is the amount of food eaten and absorbed) were about 88% for small pelagic fish and 83% for pelagic finfish. The overall losses of biological production, from fish prey to predator trophic levels, is shown by [11] in their production flow diagram to be about 90% due to respiration and excretion (loss to detritus). The approximate 90% losses, including the normal predator consumption of individual prey densities, explain the overall 10% TTE’s between trophic levels [7]. These findings support the general applicability of Eqs 4 and 5 as the proportion of the prey biological production transferred to predators to support the predator’s biological production and maintenance.

To estimate the equivalent reduction in fishing mortality, under steady-state and ecosystem-based conditions, it is assumed that the relative amount of fish catch forgone is in proportion to the biological production transferred to predators [21]. This was tested by examining the Ecopath Model results of the North Sea fishery by [25]. This showed the fish catch and F_MSY_ were directly related to the biological production of the fishery (see proportioning F_MSY_ values for multispecies fisheries in trophic levels, below). Although recruitment varies annually [26], the F_MSY_ values are used as an average reference point for estimation of average fishing mortalities for ecosystem stability and full EBFM conditions.

To estimate the TTE’s in Eqs 4 and 5 it is necessary to know how they are estimated, which is shown in the next section.

#### Relationship of trophic transfer efficiencies with trophic levels

In order to use Eqs 4 and 5 to estimate ecosystem-based fishing mortalities, it is first necessary to know the relationship between trophic transfer efficiencies and trophic levels. Estimation of ecosystem-based F_MSY_ requires knowledge of the trophic transfer efficiencies, which are not generally known for fishery management [27]. However, [28] showed there is a relatively consistent relationship between trophic levels and transfer efficiencies. In addition, the Ecopath models in [29] estimate the biological production of fish species, groups or taxa in a wide range of aquatic ecosystems for rivers, lakes, coastal lagoons, coral reefs, coastal areas and tropical shelves. The overall average of all these fishery TTE’s, estimated by the method of flows of food into a TL, from all food sources, and flow out by consumption by all predators is shown by [28] averaged about 10%. However, as stated above, the biological production transfers are only applied here to marine pelagic fisheries. The average transfer efficiency from TL2 to TL3 (zooplankton to small pelagics) was about 11.1%, TL3 to TL4 (small pelagics to predatory pelagic finfish) 8.5% and TL4 to TL5 (e.g. sharks) 7.6% with an overall average of 9.2% and standard error of 1.25%. The average TTE’s for nine of the coastal and tropical shelf marine ecosystems, shown in [28], was TL2 to TL3 14.8%, TL3 to TL4 9.4%, and TL4 to TL5 7.7%, with an overall average of 10.6%. These results indicate a decrease in efficiency as the trophic level increases.

Similar results and decreases were found for the estimated TTE’s in the twelve small pelagic and pelagic finfish Ecopath model fishery examples used to test EBFM theory (see Ecopath model fishery examples to test EBFM theory, below). The TTE for TL2 to TL3 was estimated to be 13.5 ± 3.3%, the TL3 to TL4 as 10.8 ± 3.1%, and TL4 to TL5 6.0 ± 1.2% (Tables 2 and 3). These give an overall average of 10.1%, similar to that typically reported in the literature [7]. The relationship of TTE with TL (the trophic level receiving the transfer) from TL2 to TL5 was examined by regression of TTE plotted against TL using the average of the nine marine ecosystems and from Tables 2 and 3, giving the power function:

$$TTE=0.54\;x\;TL^{‐1.26}.$$ (6)

**Fig 1:**
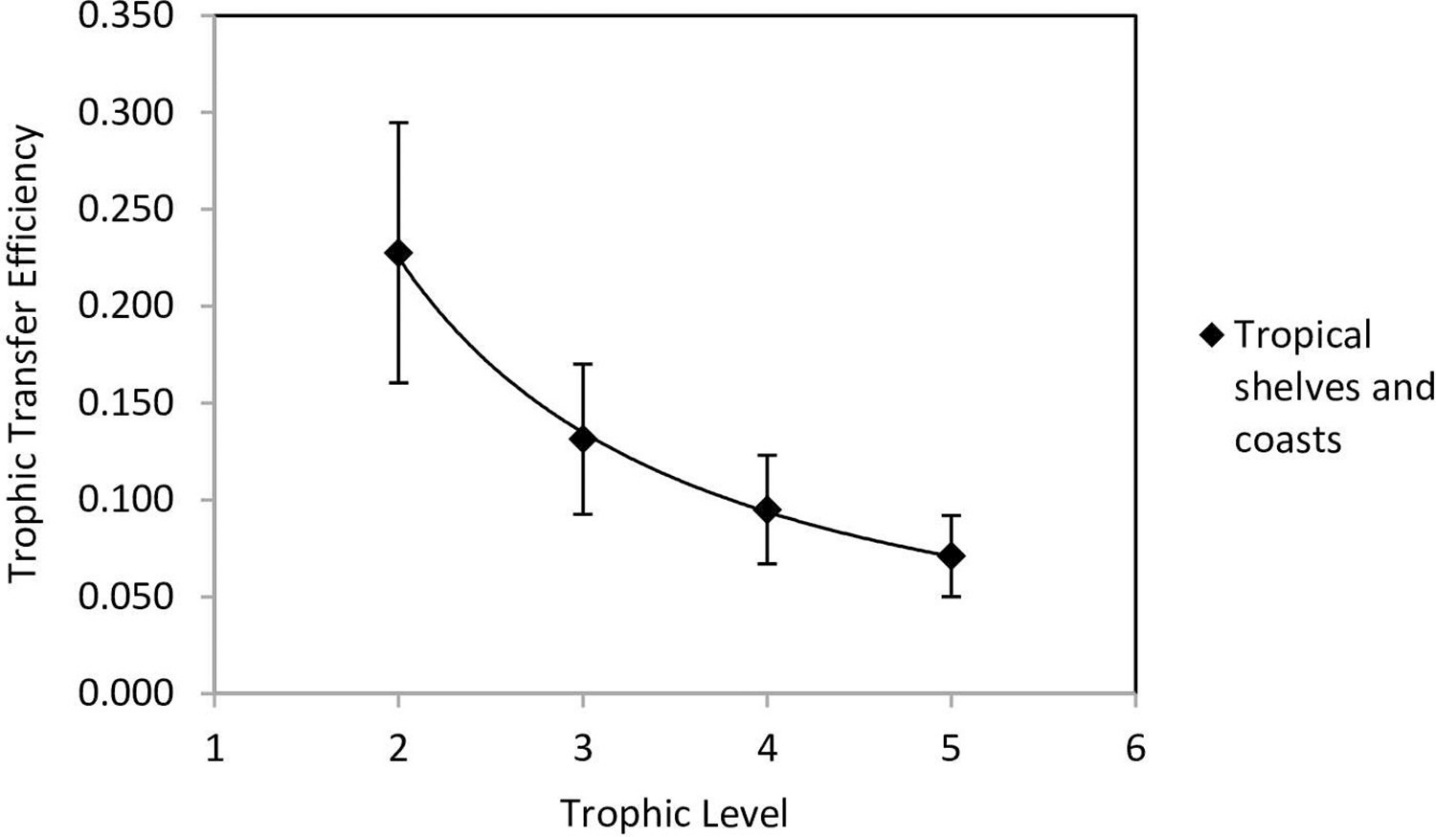
Relationship of trophic transfer efficiencies with trophic levels and standard deviation ± 0.295 of average TTE. Transfer efficiencies for phytoplankton (TL1) transfer to zooplankton (TL2) estimated by [29], (their Table 3) were at the low end of range 0.12 to 0.35 reported by [74], so TTE for the receiving TL2 was estimated by regression, giving an average of 0.228 ± 0.0067 for 1 standard deviation.

The regression was (R^2^ = 0.9477, n = 4, p <0.002) with an average standard deviation, σ ± 0.039 for the four trophic levels shown in Fig 1. Standard deviations are based on 0.295 x TTE, giving TL2 ± 0.067, TL3 ± 0.040, TL4 ± 0.028 and TL5 ± 0.021. The nine fisheries from [28] and the twelve from Tables 2 and 3 gave 45 data points to calculate the averages for each TL and error bars for each point. The error bars in Fig 1 provide fishery managers more flexibility in selecting fishing mortalities relevant to the fishery conditions. Although phytoplankton (TL1) transfer to zooplankton (TL2) is not needed for fishery management, it is of fundamental importance to fishery production. Reference [28, 29] estimated them for the nine marine ecosystems, but the TTE average was at low end of the range 0.12 to 0.35 suggested by reference [30] and did not fit the regression. The expected TL2 average was estimated from the TL3 to TL5 regression at 0.228 ± 0.067 for 1 standard deviation, giving error bars 0.16 to 0.29, which was considered acceptable and is included in Fig 1.

The relatively consistent results of Eq 6 show the estimated TTE’s could be used for fisheries where Ecopath Model biological production data, B*(P/B), for predators and prey are not available. Therefore, the estimated TTE’s could be used to estimate the proportional reductions in fish catch and equivalent fishing mortalities for EBFM conditions.

The following conditions and definitions are necessary to obtain estimates of the EBFM F_MSY_: (i) proposed F_MSY_ upper limit to provide a baseline for data-limited fisheries, (ii) precautionary factors to estimate ^SSR^F_MSY_ for spawning stock recruitment (SSR), (iii) definition of ecosystem stability. Those are followed by an explanation of the assumptions used and examples of estimating EBFM fishing mortalities.

### Proposed F_MSY_ upper limit

The review of global fisheries by [31] found over half the fisheries examined were data-limited fisheries and most were overfished because they could not judge the status of their fisheries. The characteristics of r, B_0_ and M_0_ required to model the status of a fishery are difficult to estimate [31 - page 24, 32, 33], and the data-limited fisheries apparently do not have the resources to do those calculations. Therefore, it is necessary to define an upper limit for F_MSY_ to establish a baseline for reductions to the recruitment ^SSR^F_MSY_ and reductions of ^SSR^F_MSY_ to ecosystem based F_MSY_. With the baseline, data-limited fisheries have a simple method to estimate EBFM F_MSY_ values for pelagic fisheries without undertaking complex stock assessment modelling.

Although the [34] stated the F_MSY_ is a limit rather than a target for fishing mortality, an upper limit has not been defined. However, [35] investigated the relationship MSY = 0.5MB_0_ (M is natural mortality and B_0_ projected unfished biomass) because it was considered unreliable. They concluded a preliminary maximum target between 1/2 and 2/3 of the estimated MSY (i.e., equivalent to an upper limit of F_MSY_ of 0.5 under equilibrium conditions) be used for initial considerations of fishery management. Analytically, the 0.5 limit can be tested by considering that MSY occurs at half B_0_, where the fishery biomass is increasing according to B_t+1_ = B_t_ x e^rt^, r is the intrinsic rate of natural increase and t is one year. Under these conditions, F_MSY_ = r/2 [16], equation 13.7. If the F_MSY_ upper limit is set at 0.5, then r = 1.0 and the fishery has to replace half its biomass in one year, so B_t+1_ = B_t_ x 1.5. However, e^1.0^ = 2.718, which is more than enough to replace the fishing mortality. On the other hand, [36] showed that predators consume more fish than taken by fisheries and its affect should be taken into account for fishery management. For example, [24] estimated the predator mortality, M_2_, on European anchovy, pilchards and other small pelagic fish by pelagic finfish predators, which averaged 0.50. Assuming this level of predation is typical for the highly productive small pelagic fish, the exponent is reduced by 0.5, giving e^0.5^ = 1.649 x B_t_, sufficient to replace the effects of fishing with some for M_0_ (other natural mortality).

For highly productive small pelagic fish such as herrings and sardines, r is expected to be in the range of high resilience from 0.6 to 1.5 [37]. The r value for pelagic finfish predators is expected to be lower because they have a lower average P/B ratio of 0.38 compared to 0.91 for small pelagic fish [23]. As the P/B ratio is the rate of biomass regeneration [18], the r value for the Atlantic bonito and large pelagic fish predators is expected to be in the medium resilience range of 0.2 to 1. On this basis, with the medium resilience range up to 1.0, the 0.5 upper limit for F_MSY_ is considered appropriate for both the pelagic finfish predators as well as the small pelagic fish. This baseline is supported by the F_MSY_ for North Sea fisheries. The highest F_upper_ of 0.52 was estimated as the upper limit to reduce the risk of stock collapse by [38]. Further support is provided by the recent fish mortality adjustments by [15] with implementation of ecosystem-based F_MSY_ values. They found all North Sea F_MSY_ values were <0.5, indicating an F_MSY_ > 0.5 are not advised and not sustainable in the long-term. That conclusion comes from [38] on ecosystem based F_MSY_ values for fisheries management. The report shows of the 54 fisheries examined, the ecosystem-based F_MSY_ values were less than 0.5 and only one was above 0.5 at 0.52. Hence, an upper limit of 0.5 is used here as a baseline to estimate F_MSY_ with recruitment, ^SSR^F_MSY_, by application of established precautionary factors. The resulting ^SSR^F_MSY_ are then used as Target Reference Points (TRP) for estimation of EBFM fishing mortalities. The precautionary factors applied to the F_MSY_ 0.5 baseline used to estimate the ^SSR^F_MSY_ for spawning stock recruitment are shown in the next section.

### Precautionary factors and estimation of ^SSR^F_MSY_ with recruitment

Adjustment of the upper F_MSY_ baseline of 0.5 by precautionary factors for small pelagic fish and pelagic finfish to allow for recruitment included in the ^ES^F_MSY_ calculation is necessary because the F_MSY_ was found to cause overfishing due to recruitment by the spawning stock biomass included in the calculation of MSY. The MSY is based on the increase in biomass due to the net of biomass growth + recruitment—natural mortality [16], Section 13.2.1, Page 310. Hence, the precautionary factors are used to reduce the F_MSY_ baseline to the ^SSR^F_MSY_ with recruitment. As shown below, the typical ^SSR^F_MSY_ values are estimated by reduction of the upper F_MSY_ of 0.5 baseline by precautionary factors of 2/3 for small pelagic fish and 3/4 for pelagic finfish, giving ^SSR^F_MSY_ of 0.335 for small pelagic and 0.375 for pelagic finfish. Application of precautionary factors to the upper F_MSY_ baseline means the ^SSR^F_MSY_ values include an ecosystem component, so they are called EBFM ^SSR^F_MSY_.

Fishery management strategies have adopted modification of the F_MSY_ so the resulting fishing mortalities and Total Allowable Catch (TAC) are adjusted to maintain the SSB [15]. However, for data-limited fisheries where the SSB cannot be modelled, the ^SSR^F_MSY_ with recruitment could be estimated by application of precautionary factors to the upper F_MSY_ baseline of 0.5. The FAO [34] proposed the recruitment factor 2/3 (taken as 0.67) as a target reference point because of its performance in terms of reducing risk to overfishing. It was suggested to be applied to the small pelagic fishery trophic level to obtain a sustainable F_MSY_. Consequently, it has also been widely used for other fisheries [39, 40] because F_MSY_ is a limit, rather than a target, that needed to be reduced by precautionary factors to adjust the F_MSY_ for recruitment. Hence, the [34] factor is considered acceptable owing to the work by [41, 42], who found small pelagic fish are susceptible to effects of recruitment success. Application of the 2/3 factor to the F_MSY_ upper limit gives a typical ^SSR^F_MSY_ of 0.335 (0.5 x 0.67) for small pelagic fish adjusted for spawning stock recruitment. By comparison, a reduction in F_MSY_ from a high of 0.67 to 0.32 was estimated by a dynamic pool production model used by [43].

Similarly, TL4 pelagic finfish predators tend to have their F_MSY_ reduced by 0.75 F_MSY_ as a precautionary approach for recruitment by the spawning stock biomass [44–46]. The same factor for estimation of an Acceptable Biological Catch (ABC) was used by [47]. This recruitment factor reduces the upper limit of the F_MSY_ to about 0.375 (0.5 x 0.75) and is similar to the F_MSY_ of 0.35 typically used for predatory fish [48]. As these fishing mortalities are in the range typically used to maintain multispecies fisheries [38], it is suggested that overfishing could be minimized by having the ^SSR^F_MSY_ values of 0.335 and 0.375 used as upper limit Reference Points for estimation of EBFM F_MSY_. Note that by reducing the upper F_MSY_ to allow for recruitment, the ^SSR^F_MSY_ have an EBFM component, so they are called EBFM ^SSR^F_MSY_. In addition, reductions to the ecosystem-based F_MSY_ may need corresponding reductions in fishing effort and allowance for potential changes in the catchability coefficient [49, 50], as undertaken recently by [15].

### Definition of ecosystem stability

Although there have been many studies on the stability of a fishery, there appears to be no accepted definition of ecosystem stability [51, 52]. However, [53], page 15, noted it was most likely that stable, balanced ecosystems depend on predators maintaining their prey food population by avoiding over consumption of the prey. He also noted that the predators, by only eating the annual increase of the prey population, the prey breeding stock is not put in danger and the prey continue to support the predators. Those observations indicate, to maintain stability, the predator consumes an appropriate level of prey production, thereby maintaining the prey population, which in turn supports the predator’s population and production. The importance of maintaining predators for ecosystem stability is discusses further by [54, 55] provides empirical evidence that the depletion of predatory species (by over fishing) can affect the dynamic stability of natural fish communities. Hence, the factor $\sqrt{TTE}$, by providing the proportion of the prey fishery biological production for maintenance of predators, maintains ecosystem stability. The factor is based on how natural fish communities function, where the prey biomass is maintained because predators only consume the prey biological production, as shown by the trophic transfer efficiencies in Eq 1. Note that for transfers from TL2 to TL3, zooplankton are the prey and small pelagic fish the predator, while for TL3 to TL4, small pelagics are the prey and pelagic finfish are the predator.

The importance of the relationship of predator-prey biological production transfers to ecosystem stability is also supported by Ecopath Models used for fishery management, based on the fundamentals set out by [56, 57], their master Eq 1. Their trophic mass-balance biological equations are: Biological Production = Fish catch + predation mortality + other mortality + biomass accumulation + loss to adjacent systems. Therefore, fish catch is related to biological production, fishery biomass and predator consumption. Hence, ecosystem stability is proposed to be defined as: *Ecosystem based fisheries maintain predator biological production by managing the fishing mortality to allow consumption of sufficient prey biological production to support the predator’s biological production in the next higher trophic level*. This way, the EBFM works with the natural processes of the prevailing fish community. Therefore, it is suggested progress could be made in understanding how EBFM functions from the perspective of natural fish community processes.

### Assumptions and examples for estimating ecosystem-based ^ES^F_MSY_ and ^SB^F_MSY_

In order to understand how the ecosystem-based ^ES^F_MSY_ and ^SB^F_MSY_ are estimated in the Results Section, it is necessary to first explain the assumptions and show, by examples, how the results are obtained. To begin, it is important to know that without allowing for predator-prey biological production transfers to maintain ecosystem stability, the fishing morality, including by-catch mortality, could lead to an imbalance of trophic interactions.

The following assumptions and examples show how Eqs 4 and 5 for predator-prey transfers via the trophic transfer efficiencies can be used to maintain ecosystem stability. For ecosystem stability, it is necessary to maintain predator production. Using small pelagic fish (TL3) as an example, the first step is to estimate the proportion of small pelagic production consumed by the pelagic finfish predators to support the predator production in the next higher trophic level. Hence, the trophic level used to estimate ecosystem stability is the TL_n+1_, where n is the TL of the species being fished. The TTE_n+1_ is then estimated using Eq 6 for the finfish predators TL4 of say 4.0: 0.54 x 4^−1.26^ = 0.094 and take the square root, giving $\sqrt{0.094}$ = 0.307. As 0.307 of the small pelagic production is used to maintain pelagic finfish predators, the remaining 0.693 (1–0.307) is allocated to the small pelagic fishery. In most cases, data-limited fisheries don’t know the fishery biomass, or it is badly estimated, so the remaining 0.693 is multiplied by the upper F_MSY_ baseline of 0.5 to limit the ecosystem-based F_MSY_ to <0.5. In this example, the F_MSY_ = 0.347 (0.693 x 0.5). The final step is to estimate the ecosystem stability fishing mortality, ^ES^F_MSY_ by multiplying the baseline adjusted F_MSY_ by the precautionary factor, PF, for small pelagic fish of 2/3 (taken as 0.67) for spawning stock recruitment, ^SSR^F_MSY_, giving ^ES^F_MSY_ = 0.232 (^SSR^F_MSY_ = 0.67 x 0.347). These processes are summarised in the following equation:

$${}^{ES}F_{MSY}=PF\;x\;0.5\;x\;(1‐\sqrt{TTE\;n+1)}.$$ (7)

Note that the TTE_n+1_ in Eq 7 is for the pelagic finfish predator (TL4) in the next higher trophic level above that of small pelagic fish at TL3. Therefore, the ^ES^F_MSY_ for small pelagic fish uses the TTE estimated for TL4 predators. Application of Eq 7 to pelagic finfish uses TL5 to calculate TTE_n+1_ for pelagic finfish production required to support the TL5 predator production.

To estimate the full EBFM F_MSY_ for ecosystem and biomass stability, ^SB^F_MSY_, it is necessary to protect biological production of the prey in the next lower trophic level. Hence, in this example, TL3 is used to calculate the TTE because small pelagic fish are predator of the zooplankton prey input. That is, ^SB^F_MSY_, is estimated by limiting the small pelagic fishing mortality to a proportion of zooplankton biological production entering TL3, the TL_n_ of the species or taxa being fished. The TTE for input to TL3 at say 3.0 is 0.54 x 3^−1.26^ = 0.135 and $\sqrt{0.135}$ = 0.367, which is the proportion of zooplankton production forgone to the small pelagic fishery. Hence, the ^ES^F_MSY_ of 0.232 is multiplied by 0.367, giving the ^SB^F_MSY_ = 0.085. To estimate the ^SB^F_MSY_, Eq 7 is modified to:

$${}^{SB}F_{MSY}={}^{ES}F_{MSY}\;x\;\sqrt{TTEn}.$$ (8)

Note that for small pelagic fish the TTE_n_ in Eq 8 is for TL3 because they consume production of the next lower trophic level. For pelagic finfish fishery, the TTE_n_ is for TL4, the TL_n_ of the species, groups or taxa in the same TL being fished, because they consume the small pelagic fish production.

As a further example, the following is used to show how to estimate the ecosystem stability, ^ES^F_MSY_, when the F_MSY_ for a sustainable fishery, with allowance for recruitment is known. For predatory fish, [27], their page 855, found such an F_MSY_ was 0.35. For the typical TTE of 0.10, Eq 7 shows the typical amount of F_MSY_ foregone to support the predatory fish biological production is $\sqrt{TTE}$ = 0.316. The remaining 0.684 (1–0.316) is allocated to the prey fishery. The resulting prey fishing mortality for ecosystem stability, ^ES^F_MSY_, is 0.35 x (1–0.316) = 0.24. The estimated ^ES^F_MSY_ of 0.24 is similar to the biomass based F of 0.25 shown by [28] for a sustainable long-term yield. Hence, it is proposed that ecosystem stability is expected to be achieved by supporting the biological production of predators.

## Results

To estimate the ecosystem-based ^ES^F_MSY_ for ecosystem stability, the ^SSR^F_MSY_ are reduced by the proportion of biological production forgone to support predators. Hence, the results for estimation of EBFM ^ES^F_MSY_, the corresponding reductions and comparisons with the literature, are presented.

### Fishing mortalities for ecosystem stability, ^ES^F_MSY_

The estimated trophic transfer efficiency of small pelagic biological production to predatory pelagic finfish in TL4 is 0.54 x 4^−1.26^ = 0.094 from Eq 6. From Eq 4, the proportion of small pelagic biological production to be forgone to support pelagic finfish biological production, is 30.7% ($\sqrt{0.094}$ = 0.307). From Eq 7, this proportion reduces the small pelagic catch, and related fishing mortality, below the ^SSR^F_MSY_ for recruitment of 0.335 (upper F_MSY_ 0.5 x PF 0.67). Hence, the average ^ES^F_MSY_ for ecosystem stability is 0.23 ± 0.020 (0.335 x (1–0.307)), with the range estimated from TTE ± 0.028 for TL4 in Fig 1. Likewise, the pelagic finfish fishing mortality for ecosystem stability is reduced from 0.375 to allow for TL5 pelagic predators (mostly sharks) consuming some of the PF biological production. The TTE to TL5 predators by Eq 6 is 0.071, so the proportion of PF biological production transferred is $\sqrt{0.071}$ = 0.266. For ecosystem stability, the ^SSR^F_MSY_ for recruitment of 0.375 (upper F_MSY_ 0.5 x PF 0.75) gives an expected average pelagic finfish fishing mortality, ^ES^F_MSY_ = 0.27 ± 0.026 (0.375 x (1–0.266)).

Some confidence in the method is provided by the ^ES^F_MSY_ obtained being similar to those by EBFM modellers, when the variability reported for aspects of fishery management is taken into account. This can be seen by the theoretical estimates for small pelagic and pelagic finfish being similar to those obtained by [57] of about F = 0.22 for herring and F = 0.26 for cod in the North Sea. Their fishing mortalities were obtained at optimum fishing rates predicted by their base model for EBFM with economics included. The ^ES^F_MSY_ obtained for small pelagic fish is similar to the 0.25 for herring, mostly for ecosystem benefit, obtained by [59]. The fishing mortality for herring at 0.27 to 0.28 [59] was moderately higher than the 0.22 estimated by [58, 59]. More recently, [15] has the herring F_MSY_ at 0.26 to 0.31 and as low as 0.157 (to allow for recovery from overfishing), depending on the need to maintain the SSB. The typical ^ES^F_MSY_ of 0.27 for TL4 pelagic finfish is similar to those by [60] for reduced fishing pressure on Haddock (0.3), North Sea Plaice (0.25) and Saithe (0.3). For pelagic predatory fish, [15] have the F_MSY_ at 0.26 for mackerel and 0.32 for blue whiting. These comparisons suggest the ^ES^F_MSY_ of 0.23 for SP and 0.27 for PF represents priority for the fishery ecosystem with economic considerations included.

Due to the need to account for the status of the fisheries, the variation around the expected ^ES^F_MSY_ values is similar to the average TTE standard deviation of 0.037 in Fig 1. For example, applying standard deviations for the reported fisheries gives 0.23 ± 0.03 for small pelagic fish (not including overfishing effects) and predatory fish 0.27 ± 0.026. Hence, modelling for EBFM could include the ecological processes for ecosystem stability indicated by the ^ES^F_MSY_ of 0.23 to 0.27 with practicable ranges of about ± 0.03. This is supported by the findings of [61], who found global fisheries could be rebuilt with a fishing mortality of about 0.25, giving a sustainable long-term yield.

To estimate the ecosystem-based F_MSY_, with most emphasis on the fishery ecosystem, the ^ES^F_MSY_ is reduced to give the full EBFM for ecosystem stability and biomass, ^SB^F_MSY_. The results, corresponding reductions and comparisons with the literature are presented next.

### Fishing mortalities for ecosystem stability and biomass, ^SB^F_MSY_

As shown above, a fishery operated for ecosystem stability provides food for predators in the next higher trophic level, but for full pelagic EBFM conditions, the biological production of prey in the next lower TL also has to be maintained. From Eq 6, the TTE of zooplankton biological production entering the small pelagic fish trophic level in TL3 is 0.54 x 3^−1.26^ = 0.135. From Eq 5, the proportion of incoming prey, the zooplankton biological production, to be forgone is 36.7% ($\sqrt{0.135}$ = 0.367). As shown in Eq 8, the small pelagic fishery is the receiver of the zooplankton biological production, the SP fishing mortality for ecosystem stability, ^ES^F_MSY_ of 0.23, is further reduced by 36.7%. Therefore, the expected average ^SB^F_MSY_ is 0.084 ± 0.011 (0.23 x 0.367, range estimated from TTE ± 0.04 from Fig 1).

The full EBFM F_MSY_ for the TL4 pelagic finfish fishery is estimated in a similar way by limiting the fishing mortality to a proportion of small pelagic, TL3, biological production entering the pelagic finfish TL4 trophic level. The TTE biological production transfer from TL3 to TL4 is 0.094 (Eq 6), so the pelagic finfish ^ES^F_MSY_ of 0.27 is reduced by 30.7%: $\sqrt{0.094}$ = 0.307, giving an average pelagic finfish fishery ^SB^F_MSY_ of 0.083 ± 0.012 (0.27 x 0.307). These EBFM ^SB^F_MSY_ values provide for ecosystem stability with support for the fishery biomass. Hence, the relatively low fishing mortalities support the biological production and biomass of both the small pelagic prey and the pelagic finfish predators. That is, by including ecological processes of biological production, biomass and ecosystem stability, the full EBFM ^SB^F_MSY_ gives an ecosystem-based fishery providing for the needs of prey and predator in proportion to their productivity.

The EBFM ^SB^F_MSY_ values are similar to the low fishing mortalities of 0.05 to 0.10 to ensure sustainability of the Australian small pelagic fishery and recovery from overfishing [62]. As well, a fishing mortality of 0.10 was proposed as a sustainable harvest rate for a long-lived tropical fish [63]. The potential target reference points for rebuilding stocks was reviewed by [64] and noted heavily depleted stocks increased when the fishing mortality was less than 0.2. Recently, [15] has set horse mackerel F_MSY_ = F_pa_ (precautionary fishing mortality for SSB) to allow recovery from overfishing using a fishing mortality of 0.074 to 0.079. To allow for practicable considerations for fishery management, applying standard deviations for the reported fisheries gives the EBFM ^SB^F_MSY_ values of about 0.084 ± 0.02.

### Proportioning average F_MSY_ values between multispecies fished in a trophic level

The fishing mortalities are equivalent to a single-species F_MSY_ averaged over the catchable species fished in a trophic level. Hence, the multispecies fishing mortalities for each species harvested inside a trophic level is estimated by adapting the method of [65] by distributing the F_MSY_ according to the catch for each species relative to the average historical catch. As an example, multispecies fishing mortalities are proportioned by their fish catch using the basic fishery characteristics from the Ecopath Model of the North Sea fishery by [24], shown here in Table 1.

**Table 1:** Proportions of multispecies F_MSY_ and catches between species fished in a trophic level.

Species	Biological Production, P_exist_, (t/km^2^/year)	Existing Fish Catch, FC_exist_, (t/km^2^/year)	Existing Biomass, B_exist_, (t/km^2^)	F_exist_	Proportion F_msy_ by FC for average F_MSY_ = 0.27	Proportion F_msy_ by P for average F_MSY_ = 0.27	Estimated FC using F_msy_ by FC (t/km^2^/year)	Estimated FC using F_msy_ by P (t/km^2^/year)
Adult Cod	0.192	0.124	0.161	0.770	0.343	0.312	0.054	0.052
Whiting (adult)	0.313	0.181	0.352	0.514	0.501	0.509	0.079	0.084
Saithe (adult)	0.209	0.117	0.220	0.532	0.324	0.340	0.051	0.056
Hake	0.011	0.005	0.014	0.357	0.014	0.018	0.002	0.003
Blue Whiting	0.105	0.061	0.042	1.452	0.169	0.171	0.027	0.028
Average	0.166	0.0976	0.158	0.725	0.270	0.270	0.043	0.045

This method of estimating multispecies F_MSY_ is not for data-limited fisheries because they most likely obtain an acceptable fishing mortality using single-species ^ES^F_MSY_ and ^SB^F_MSY_ values. The multispecies procedure requires the estimated EBFM ^ES^F_MSY_ by the method developed here, such as 0.27 used in Table 1, and the following knowledge of a managed fishery or from an Ecopath Model of the fishery: (i) catch of all the species caught, (ii) average fish catch of all the species caught in the fished trophic level, (iii) average biomass for all the species caught. For example, adult cod in Table 1 has the FC 0.124 t/km^2^/year, average fish catch for the trophic level 0.0976 t/km^2^/year, and average biomass 0.158 t/km^2^. Using the EBFM ^ES^F_MSY_ of 0.27, the adult cod EBFM F_Msy_ = 0.27 x 0.124/0.0976 = 0.343 and the FC = 0.343 x 0.158 = 0.054 t/km^2^/year. In this way, the EBFM F_Msy_ and catch is proportioned for each species in a multispecies fishery, as shown in Table 1.

At the time of running the model in 2007, the average F_MSY_, F_exist_, was 0.725 and average catch 0.098 tww/km^2^/year for five predatory species with most of their diet as small pelagic fish [24]. Assuming F_exist_ applies to TL4, the average catch was reduced to the EBFM ^ES^F_MSY_ of 0.27 for pelagic finfish (estimated in the Results Section). Portioning of the fishing mortalities was undertaken using the 2007 catches by the equation: F_Msy *i*_ = 0.27 x (FC_species *i*_ / average FC_all species_). The multispecies F_MSY_ values were estimated using the average FC_exist_ because the ^ES^F_MSY_ is the average of the species fished in the TL. The resulting proportions of the F_Msy_ values are shown in Table 1. Fish catches were estimated by setting the traditional biomass F = proportioned F_msy_ by: FC_*i*_ = F_species *i*_ x B_average_, where the average fishery B_average_ was taken as the 2007 0.158 t/km^2^. Note, the method should not be used for mixed fisheries, spread over more than one trophic level, because the average F_MSY_ values apply to only one trophic level.

**Fig 2:**
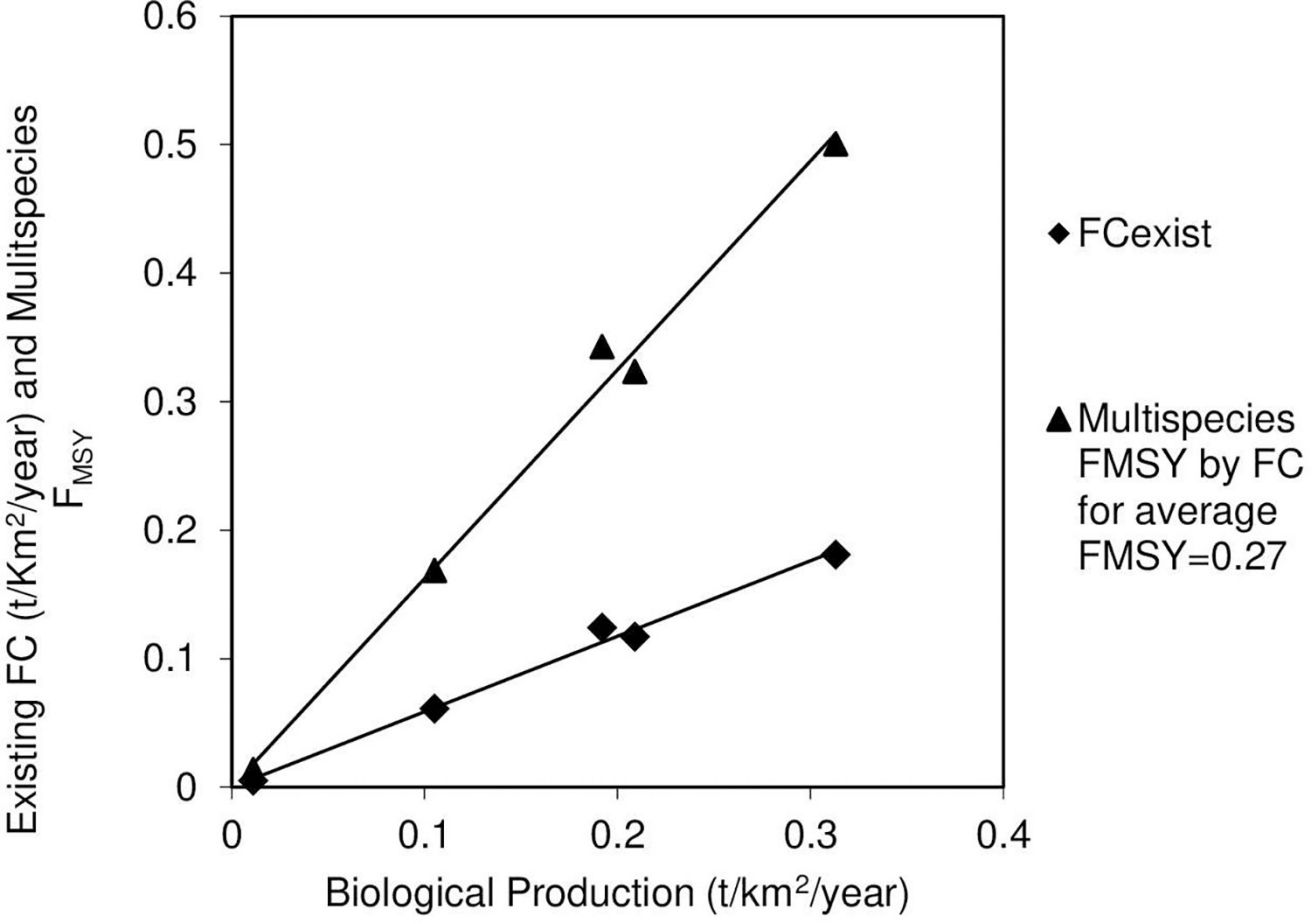
Relationship of fish catch (t/Km^2^/year) and multispecies F_MSY_ with biological production within a trophic level.

For comparison, multispecies fishing mortalities are also proportioned by their biological production because the catches are directly related to biological production by FC_exist_ = 0.5872 x P_exist_ (R^2^ = 0.9905, n = 5, p <0.001 intercept set to 0,0), Fig 2. Significant relationships between catch and production were also observed for tropical small pelagic fish and their pelagic finfish predators in the section on Ecopath model fishery examples to test EBFM theory, below.

Fig 2 shows the multispecies F_MSY_ are also related to the biological production, P, by F_MSY_ = 1.6245 x P_exist_ (R^2^ = 0.9905, n = 5, p <0.001). Hence, the average trophic level F_MSY_ could be proportion by biological production estimated for each species using the basic Ecopath Model P/B ratios multiplied by the existing biomass for each species fished, that is, P = B x (P/B). Table 1 shows Proportioning of F_MSY_ by biological production gave similar results to that using catch and biomass data, demonstrating biological production could also be used to estimate the multispecies catch inside a trophic level. The biological production results were obtained by assuming the species’ biological production is the 2007 P_exist_. The above FC equation was modified to proportion the F_MSY_ by P_exist_ by: F_Msy *i*_ = 0.27 x (P_species *i*_ / average P_all species_). The resulting F_MSY_ values are similar to those using fish catch because catch and biological production are related. Following the above method, catches were estimated by multiplying the biological production proportioned F_Msy *i*_ by the average P_exist_ of 0.166 t/km^2^/year by: FC_*i*_ = F_Msy *i*_ x P_average_.

#### Ecopath model fishery examples to test EBFM theory

The above relationships between fish catch and F_MSY_ shows, under ecosystem based conditions, the F_MSY_ or the biomass F are expected to be related to the biological production of the fishery. This is examined by using published Ecopath Models for tropical shelves and coasts where ecosystem-based fisheries are expected to exist.

To describe new knowledge and insights how EBFM might work in practice [10] also suggested using an example of published fishery data. The Ecopath Model estimates the biological production transfers from phytoplankton to zooplankton, then into the pelagic finfish ecosystem, for each trophic level. Preference for each predator to consume each type of prey in a trophic level is shown in the diet matrix tables. An example is the Venezuelan fishery diet matrix [66], Table 4, page 292. The Ecopath Model [11], their production flow diagram, together with [67], their flow diagram, shows the opportunist consumption of alternate food sources by predators during temporary shortage of the more abundant preferred prey. The limited food energy obtained by consumption of alternate prey is used for maintenance of respiration and excretion by the predators. The more abundant preferred prey, in the next lower trophic level, also has most of their food energy used for predator maintenance, but with sufficient left over for growth of the predators. This way, about 10% of the preferred prey biological production is transferred to predator biological production in the next higher trophic level. Hence, TTE’s are estimated by the ratio of predator to prey biological production between trophic levels [7]. Accordingly, published Ecopath Model results in [27] for biological fish production of small pelagic fish and their pelagic finfish predators at tropical shelves and a coastal fishery, with their associated fish catch data, were used to help understand what pelagic EBFM fishing mortalities actually are in practice.

The approach follows the suggestion by [58] that there is a need for direct empirical evidence on the relationships between forage fish and their predators to assist modelling for fishery management, taking into consideration that some of the empirical data may be unreliable. Accordingly, as for all models, the results were checked for consistency with the model inputs, see [19]. The published Ecopath Model results used are four in the Gulf of Mexico: coastal fishery in South Western Gulf of Mexico, SWGoM, [68], shelf fisheries at Gulf of Mexico, GoM, [69], Yucatan, YUC, [70] and Venezuela, VEN, [66], as well as two from a wider geographic range, one at Brunei Darussalam, BD, on the NW coast of Borneo [71] and one at the Australian North West Shelf, NWS, [72]. This grouping was used to help detect unreliable data by comparison of the results located in close proximity in the Gulf of Mexico, with the results of the two fisheries further away. The NWS study was included to assist with this investigation because the pelagic fish are essentially unfished [72]. If the biological production in that area was similar to, or related to that in the other five, it may confirm what an ecosystem-based fishery is in practice. The six fisheries were selected on the following basis: (i) not managed by the traditional Maximum Sustainable Yield (MSY) at the time of the Ecopath modelling in 1993, (ii) had relatively high biomass and biological production, (iii) fish catches are related to the biological production and (iv) fishing mortalities lower than in fisheries that traditionally only provide for recruitment. The reason for selecting tropical shelves and coasts was based upon the assumption that fishing effort may decrease once there was a significant decrease in the fishery biomass, causing an increase in fishing costs [73]. A limit to fishing could be expected in fisheries, operating in developing countries, if the increased costs could not be passed on to consumers. Under these conditions, an ecosystem-based fishery may exist that could be used to test for new knowledge relating to such fisheries.

The Ecopath Model results for the above six fisheries are shown in Tables 2 and 3. Please note, to show the connection between small pelagic fish and pelagic finfish, the TTE of small pelagic input to the pelagic finfish production, Ppf/Psp, is shown in Table 3.

**Table 2:** Small pelagic tropical shelf fishery characteristics for TTE, P/B, biological production (tww/km^2^/year), biomass and fish catch (tww/km^2^/year) and fishing mortality and exploitation rate.

Fishery	Zooplankton Production (Pz^c^) input to Small Pelagic fishery	TTE Zooplankton input to Small Pelagic Production Psp/Pz	Small Pelagic Biological Production (Psp)	Biomass (Bsp)	P/B	Fish Catch, FC	Fishing Mortality F = FCsp/Bsp	Exploitation Rate E = FCsp/Psp
GoM	142.8	0.145	(6.5) **20.75**^	12.5	(0.52) **1.66**^	1.310	0.105	0.063
YUC	(150.5) **30.1**^**a**^	0.194	5.83	5.3*	1.10	0.085 [0.233]	0.016 [0.044]	0.015 [0.040]
SWGoM	229.6	0.130	(38.3) 29.75*	13.8*	(2.775) 2.156*	0.015 [2.042]	0.001 [0.148]	0.0004 [0.069]
VEN	326.4	0.115	37.63	33.30	1.130	2.667	0.080	0.071
BD	(164.5) **32.9**^a^	0.130	4.29	1.812	2.370	0.283	0.156	0.066
NWS	(1200) **240**^b^	0.097	23.25	11.34	2.050	0[*1*.*550*]	0 [0.137]	0 [0.067]
Average	(348.9) **167.0**	0.135±0.033	(19.30) **20.25**	13.01	(1.658) **1.744**	0.727 [1.348]	0.060 [0.112]	0.036 [0.063]

^Author stated P/B of 0.52 lower than expected so adjusted using average of other fisheries (not including SWGoM) giving a Psp of 20.75 tww/km^2^/year. Both P/B and small pelagic production similar to that by [74] Geers et al. (2016).

* Author stated B and P/B not known. Adjusted B from dry wt to wet wt by divide 0.2. Anchovy P/B not consistent with other fisheries so used average P/B giving

overall P/B of 2.156 and Psp of 29.75 tww/km^2^/year.

a) Pz 164.5 too high relative to Psp 4.29 tww/km^2^/year. Appears used dry weight for zooplankton biomass so adjusted P/B x 0.2 gave 13.4 and Pz 32.9 tww/km^2^/year. Same 0.2 applied to YUC.

b) Pz 1200 too high relative other fisheries. Appears used dry weight for zooplankton biomass so adjusted P/B x 0.2 gave Zp 240 tww/km^2^/year and agrees other fisheries.

c) Pz is related to phytoplankton production, PP, at: GoM 1192, YUC 362, SWGoM 1359, VEN 3150, BD 182.5, NWS 1680, average 1321 (tww/km^2^/year).

**Table 3:** Pelagic finfish fishery tropical shelf characteristics for TTE, P/B, biological production (tww/km^2^/year), biomass and fish catch (tww/km^2^/year) and fishing mortality and exploitation rate. Finfish production input to TL_5_ TTE also shown.

Fishery	TTE Small Pelagic input to Pelagic Finfish Production Ppf/Psp	Pelagic Finfish Biological Production (Ppf)	Biomass (Bpf)	P/B	FC	Fishing Mortality F = FCpf/Bpf	Exploitation Rate E = FCpf/Ppf	TTE Pelagic Finfish input to TL5 Production P_TL5_/Ppf
GoM	0.089	(1.33) **1.846**^	(0.22) **2.987**^	(1.387)**0.618^**	(0.07) 0.350*	0.117	0.190	0.067
YUC	0.162	0.944	1.40	0.674	0.195	0.139	0.207	0.051
SWGoM	0.098	2.923	3.91	0.747	0.558	0.143	0.191	0.049
VEN	0.111	4.163	8.77	0.475	0.685	0.078	0.165	0.073
BD	0.071	0.303	0.505	0.600	0.058	0.115	0.191	0.072
NWS	0.117	2.720	4.570	0.595	0 [0.482]	0 [0.105]	0.001 [0.177]	0.047
Average	0.108±0.031	(1.918) **2.150**	(3.23) **3.69**	(0.849) **0.618**	0.261 [**0.388**]	0.132 [0.116]	0.158 [0.187]	0.060±0.012

^ B lower than expected relative to other fisheries so adjusted from dry wt to wet wt by divide 0.2. P/B higher than expected relative to others so used average of other five fisheries, giving adjusted B and Ppf of 1.846 tww/km^2^/year. P/B similar to that by Geers et al. (2016) for piscivores.

*FC lower than expected relative to other fisheries so adjusted from dry wt to wet wt by divide 0.2.

Adjustments in Tables 2 and 3 are shown in bold, mostly due to use of dry weight rather than wet weight units for biomass and biological production and its effect of P/B ratios. Adjustments made for consistency with other fisheries are explained in the notes to each table. The small pelagic catch at SWGoM appears under fished, relative to the biological production, and there was essentially no fishing at the NWS (0.0023 tww/km^2^/year, [72]. Hence, the potential small pelagic catch for NWS and SWGoM was estimated by the relationship between SP fish catch and biological production for the other four fisheries using the data shown in Table 2: FC_SP_ = 0.0756 x P_SP_—0.2075 (r^2^ = 0.9894, n = 4, p <0.001). It was also noted that the YUC small pelagic fishing mortality of 0.016 was low compared to the other fisheries, indicating under fishing, so the potential catch was estimated using the same regression (see square brackets in Table 2). The pelagic finfish catch at NWS was also essentially nil, being only 0.0023 tww/km^2^/year, so the potential NWS pelagic finfish catch was estimated by the relationship between PF fish catch and biological production for the other five fisheries using the data shown in Table 3: FC_PF_ = 0.1648 x P_PF_ + 0.0338 (r^2^ = 0.9859, n = 5, p <0.001) and is shown in square brackets in Table 3.

As well as the regressions of fish catch with biological production, the catch relationship with fishery biomass, using the data shown in Table 2, is: FC_SP_ = 0.0792 x B_SP_ + 0.3177 (r^2^ = 0.8105 n = 6, p <0.01) and FC_PF_ = 0.0741 x B_PF_ + 0.1147 (r^2^ = 0.8525, n = 6, p <0.01), using the data in Table 3. Due to these results, the fishing mortality was tested for relationship with the P/B ratio for small pelagic fish: F_SP_ = 0.0784 x P_SP_/B_SP_—0.0252 (R^2^ = 0.9365, n = 6, p <0.001), Table 2 data, and for pelagic finfish by F_PF_ = 0.255 x P_PF_/B_PF_—0.0415 (R^2^ = 0.9457, n = 6, p <0.001), using Table 3 data. As the P/B ratio is rate of biomass regeneration, these relationships show relatively low proportions of the rate of regeneration allocated to the fishing mortalities at ≈ 8% for SP and ≈ 26% for PF. This indicates the fisheries were not overfished at the time of Ecopath modeling in 1993.

As expected from these results, the fishery characteristics in the tables met or are similar to those proposed by [75] as having EBFM conditions. They proposed EBFM could have biomass landing mortalities <0.1, landings <1 tonne km^2^/year, and landings per primary production <0.001 (<0.1%). The fishing mortalities in Tables 2 and 3 are slightly higher than the <0.1 landing mortalities and the theoretical full F_MSY_ for SP and PF of about 0.08. The actual small pelagic average fishing mortality of 0.060 was <0.1 but with adjustments for potential catches was slightly higher at 0.112 (Table 2). The average mortality for pelagic finfish was greater than 0.1 but with the estimated NWS included it was 0.116 and similar to that for the small pelagic fish. The higher fishing mortalities than estimated by theoretical considerations, particularly for the more cost-effective pelagic finfish fishery, appear due to application of commercial fishing efforts to prevailing EBFM conditions.

Prevailing EBFM conditions are shown by the relationship to fish catches and primary production. The actual SP catches of 0.727 were <1, but with potential catches by SWGoM and NWS averaged 1.348 tonne km^2^/year (note no discards were reported for the fisheries). Although the PF fishing mortalities were >0.1, catches were much <1 at 0.261 or 0.388 including NWS. Phytoplankton production for the six fisheries is shown in the notes for Table 2 and averaged 1321 (t/km^2^/year). The average, actual SP catch of 0.727 tonne km^2^/year at 0.055% was <0.1% of the primary production proposed as EBFM conditions. With potential catches included it averaged 0.1%. The pelagic finfish catch was 0.02 to 0.03% of the primary production. Hence, the fishery is indicated as having EBFM conditions by the catch levels, or moderately higher with commercial fishing imposed, and EBFM conditions in proportion to the primary production. Hence, the limited increase in catch, above proposed EBFM conditions, indicates that some moderating effects by the economic constraints to overfishing appear to have been operating [76].

Those comparisons with EBFM conditions are confirmed by the relationships of fish catch with phytoplankton production, PP. The phytoplankton production for each fishery is shown in note (c) of Table 2. The small pelagic regression of catch with PP is FC_SP_ = 0.001 x PP (R^2^ = 0.8433, n = 6, p <0.01) and pelagic finfish FC_PF_ = 0.0003 x PP (R^2^ = 0.7304, n = 6, p <0.05 with intercepts 0,0), showing the importance of primary production to these tropical fisheries. In addition, the relationship of zooplankton production, Pz, with phytoplankton production was significant: Pz = 0.1064 x PP + 26.451 (R^2^ = 0.8976, n = 6, p <0.002) and supports including the transfer efficiency from phytoplankton to zooplankton in Fig 1. Hence, phytoplankton production is suggested to be considered as part of the fishery status when estimating EBFM fishing mortalities, as proposed by [77, 78].

**Fig 3:**
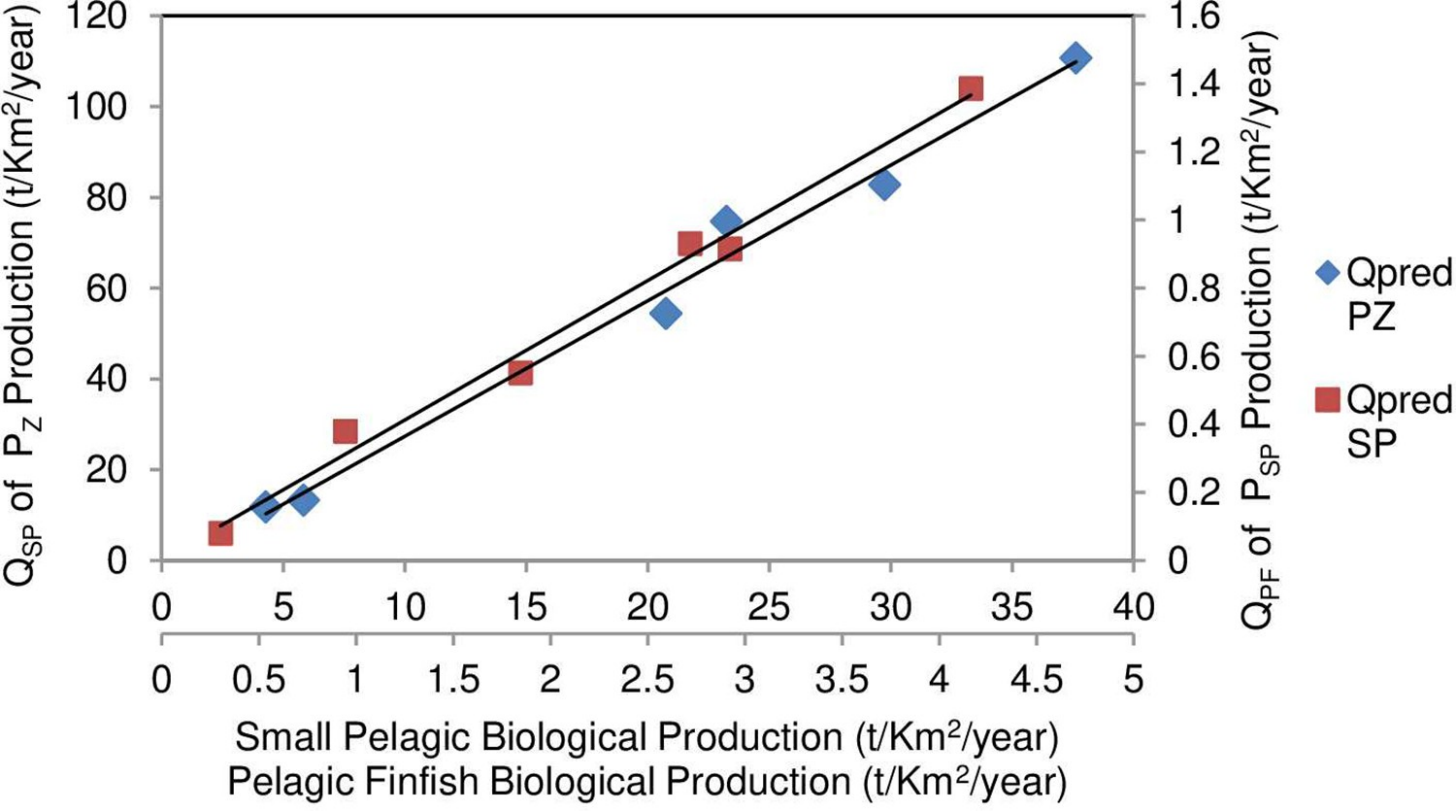
Relationship of consumption of zooplankton production, P_Z_, by small pelagic fish Q_SP_ = P_Z_ x √TTE by √(P_SP_/P_Z_) from Table 2 (LHS Y-axis), with small pelagic fish biological production, P_SP_ (upper x-axis), giving Q_SP_ = 2.9866 x P_SP_—2.5271 (R^2^ = 0.9865, n = 6, p <0.001). Similarly, consumption of small pelagic fish production, P_SP_, by pelagic finfish Q_PF_ (RHS Y-axis) and the resulting pelagic finfish biological production, P_PF_ (lower x-axis) from Table 3 has Q_PF_ = 0.3278 x P_PF_ + 0.0027 (R^2^ = 0.9888, n = 6, p <0.001).

The consumption of prey production with predator production by Eqs 4 and 5 are applied to the data in Tables 2 and 3, the relationships are shown in Fig 3.

The results show linear relationships between consumption of prey biological production and the predator production, confirming the utility of Eqs 4 and 5 for estimation of ecosystem-based fishing mortalities using trophic transfer efficiencies for ecosystem stability and support of the fishery biomass.

#### Ecosystem-based ^ES^F_MSY_ and ^SB^F_MSY_ for tropical shelves and coasts

As the above comparisons indicate ecosystem-based conditions were basically present, the ecosystem-based ^ES^F_MSY_ and ^SB^F_MSY_ for tropical shelves and coasts are estimated using Eqs 7 and 8. The TTE for small pelagic input to TL4 pelagic finfish predator production averaged 0.108 (Table 3), then from Eq 7, the small pelagic ^ES^F_MSY_ = 0.225 (0.67 x 0.5 x (1–0.329)), from ($\sqrt{0.108}$ = 0.329). The range was relatively small from 0.200 for YUC to 0.246 at BD. Those ecosystem stability fishing mortalities are higher than the estimated applied average F of 0.112, indicating a limitation on small pelagic catch. Accordingly, it is compared with the Full EBFM of ^SB^F_MSY_. Eq 8 gives the average ^SB^F_MSY_ by the TTE of zooplankton input to the small pelagic production at 0.135 (Table 2), so ^SB^F_MSY_ = 0.083 (^ES^F_MSY_ of 0.225 x $\sqrt{0.135}$ = 0.367), range 0.060 BD to 0.091 at Yucatan. As expected, the ^SB^F_MSY_ values are within the range of the actual and estimated average fishing mortalities of 0.060 to 0.112, indicating EBFM conditions.

Similarly, the average ^ES^F_MSY_ for pelagic finfish is given by the average TTE for pelagic finfish input to TL5 predator production of 0.060 (Table 3). As ($\sqrt{0.060}$ = 0.245, ^ES^F_MSY_ = 0.283 (0.75 x 0.5 x (1–0.245), range 0.274 for Venezuela to 0.294 at NWS. The pelagic finfish ecosystem stability fishing mortalities are also higher than the average applied F of 0.132. For comparison, the average ^SB^F_MSY_ is estimated by the TTE for small pelagic to pelagic finfish of 0.108 by ^SB^F_MSY_ = 0.093 (^ES^F_MSY_ of 0.283 x $\sqrt{0.108}$ = 0.329). The range of 0.075 at BD to 0.114 at Yucatan is in the range of applied fishing mortalities of 0.078 to 0.143 (Table 3), indicating the finfish fisheries were also operating as ecosystem based fisheries.

#### Ecosystem-based ^ES^F_MSY_ and ^SB^F_MSY_ for temperate fisheries

Recently, ecosystem-based fisheries management has been used to define sustainable fishing mortalities in European and other fisheries. By this process, the fisheries of each species in Table 1, for adult Cod to Blue Whiting, have had their fishing mortalities reduced since 2007 by [15] in the Greater North Sea ecoregion. They are compared with the tropical ecosystem-based ^ES^F_MSY_ and ^SB^F_MSY_ values. To provide a broader range of fished species and ecosystems, the following fisheries have been included to have their ^ES^F_MSY_ and ^SB^F_MSY_ values estimated: Mackerel (Scomber scombrus) in the Bay of Biscay and the Celtic Sea [79], European anchovy (Engraulis encrasicolus) in Northern and Central Adriatic Sea [24], Sardine (*Sardinops sagax*) in the Northern Humboldt Current Ecosystem [80] and Pacific Herring (Clupea pallasii) [81] in the Strait of Georgia Ecosystem. The ^ES^F_MSY_ and ^SB^F_MSY_ values fisheries have been estimated from their trophic levels to transfer efficiencies by Eq 6 and using Eqs 7 and 8. The results are shown in Table 4 and the applied F values reported in Ecopath Models for Mackerel to Pacific Herring are also shown in Table 4. The Sardine and Pacific Herring fisheries are included as examples of using relatively high fishing mortalities to show the expected lower ^ES^F_MSY_ and ^SB^F_MSY_ values estimated using the ecosystem-based procedure developed here.

**Table 4:** Estimated ecosystem-based ^ES^F_MSY_ and ^SB^F_MSY_ values for temperate species. Trophic levels (TL) are for the fished species and their predators in the next higher TL, along with trophic transfer efficiencies (TTE).

Species	F_MSY,_ applied F^a^	Species TL	Predator TL^c^	Predator TTE	^ES^F_MSY_	Prey input to fished species TTE	^SB^F_MSY_
Adult Cod	0.162	4.83	5.13	0.069	0.276	0.074	0.075
Whiting (adult)	0.172	4.4	4.93	0.072	0.274	0.083	0.079
Saithe (adult)	0.363	4.36	4.94	0.072	0.274	0.084	0.080
Hake	0.28	4.71^b^	5.11	0.049^d^	0.292	0.077	0.081
Blue Whiting	0.32	4.1	4.88	0.073	0.274	0.091	0.083
Mackerel	0.335	3.67	4.50	0.081	0.238	0.105	0.077
European anchovy	0.29	3.05	4.11	0.091	0.233	0.132	0.085
Sardine	0.421	3.16	4.25	0.087	0.235	0.127	0.084
Pacific Herring	0.451	3.20	4.00	0.094	0.231	0.125	0.082

a) F_MSY_ for Adult Cod to Blue Whiting from [15] and applied F reported in Ecopath Models for Mackerel to Pacific Herring.

b) From [79].

c) Adjusted Cod to Blue Whiting predator trophic level to average 5.0.

d) Due to low biomass, TTE for Hake reduced from 0.0744 by 0.295 x TTE from Fig 1.

The TL4 species in Table 4 are adult Cod to Blue Whiting and the TL3 species are Mackerel to Pacific Herring. To provide an indication of the typical range in trophic levels to allow for when estimating ^ES^F_MSY_ and ^SB^F_MSY_ values, the TL’s for the species fished and their TL4 predators are shown in Table 4. The species fished TL4 for adult Cod to Blue Whiting averaged 4.48 ± 0.29 and their LT5 predators 5.0 ± 0.11, while the species fished TL3 for Mackerel to Pacific Herring was 3.27 ± 0.27 and their TL4 predators 4.22 ± 0.22. That is, it is only necessary to know or estimate the trophic levels of the species being fish and their predators to estimate ^ES^F_MSY_ and ^SB^F_MSY_ values.

The average F_MSY_ for the five Greater North Sea ecoregion TL4 species was 0.259 but with a wide range from 0.162 to 0.363.The estimated ecosystem stability ^ES^F_MSY_ for those species has a similar average 0.278 but with a narrow range. Note that for Hake, the TTE was reduced to the low range indicated in Fig 1 due to its low biomass of 0.014 t/Km^2^, giving ^ES^F_MSY_ of 0.292, similar to the 0.28 used in [15]. The ^ES^F_MSY_ values are similar to the 0.283 ± about 0.01 for the above TL4 tropical fisheries. The applied fishing mortalities for TL3 species in Table 4 average 0.374, with a range of 0.29 to 0.451, while the ^ES^F_MSY_ for those species averaged 0.234 and similar to the 0.225 ± about 0.023 for the tropical TL3 fisheries. To provide for biomass and ecosystem stability, the ^SB^F_MSY_ values are also shown in Table 4 for the temperate fisheries and average 0.080 for TL4 and 0.084 for TL3 fisheries. They are comparable to the tropical TL4 0.093 and TL3 0.083 values. These comparisons show the procedure of mimicking the way natural fish communities function for maintaining ecological processes of biomass and ecosystem stability applies to the trophic transfer efficiencies in relatively a wide range of marine fisheries that tend to prey on and are preyed upon by pelagic fish, and in the case of TL3 species, mainly prey on zooplankton.

## Discussion

Marine ecology theory has shown how ecosystem-based fisheries could mimic natural fish community processes and thereby assistance EBFM modelling to maintain ecosystem stability and the fishery biomass. Encouragingly, the estimated fishing mortalities are similar to those reported recently for ecosystem-based fisheries with typical F_MSY_ values of 0.35 with recruitment, 0.25 for sustainable fisheries and <0.1 for full EBFM. This shows the theoretical approach has some validity in practical application to EBFM and could be used as a simple method for data-limited fisheries. In addition to the EBFM fishing mortalities being similar to those modelled, the observations of fishing mortalities for pelagic fish ecosystem stability are consistent with the Ecosystem Approach to Fisheries (EAF) proposed by the FAO to provide equilibrium between ecosystem processes and economic (fish catch) considerations [12]. Therefore, it is suggested that the ^ES^F_MSY_ be further investigated and modelled as a possible level of sustainable fish catch that allows for EBFM conditions.

When the fishing mortality of a pelagic fishery uses the full EBFM ^SB^F_MSY_, for ecosystem stability and biomass, it makes the fishery another predator integrated into the fish community ecosystem. However, this understanding of EBFM, with a fishing mortality of about 0.08, has limited consideration for economics. Hence, it could be used for recovery of overfished fisheries that require a low fishing effort to allow time for recovery. It is suggested that when a fishery is in need of recovery, fishing is not stopped but the ^SB^F_MSY_ be applied to provide fishery biomass and catch data for assessment of the fishery status as it recovers. Importantly, the data may provide an estimate of the time to recover to some benchmark below the carrying capacity (e. g. see [82]). In this regard, fishing could recommence when the biomass increases to give a sustainable ^ES^F_MSY_. This may require further research on the time for a fishery to recover with application of the full EBFM F_MSY_.

In modelled fisheries, application of ecosystem conditions to F_MSY_ values modified for recruitment, could vary the catch from the ^SSR^F_MSY_ of about 0.335 to 0.375, to the sustainable level, or to the full EBFM F_MSY_ for protection of important areas and/or recovery from overfishing. The EBFM F_MSY_ selected may need to include the results of the multispecies assessments and allowance for status of the fisheries. The proposed method for multispecies assessments is simple and could be used for managed fisheries as well as data-limited fisheries. The fishery status that provides for an appropriate level of F_MSY_ could be undertaken by investigations and modelling, such as those used by [15, 83]. Additionally, the literature and insights from the six fisheries examined shows stock status could include the ability of phytoplankton production to support the fishery.

Precautionary fishing mortality, F_pa_, reference points are used by [15] for the spawning stock biomass and most biomass based fishing mortalities are analytically assessed to keep them at or below F_MSY_. Their policy decisions also take into account species biological interactions such as predator consumption of the species fished in a stock, as well as the effects of fishing on predators and prey species. In this regard, the study here shows the importance of maintaining biological production and biomass of the small pelagic fish because they are the main food source for the prized pelagic finfish. Hence, fishing above the ^SSR^F_MSY_ for small pelagic fish could indicate overfishing, see [13], and provide an early warning for further investigation. The approach of modifying the reference point single-species ^SSR^F_MSY_ for recruitment with ecosystem-based analytical factors, allowing estimation of trophic level fishing mortalities, is suggested to be investigated for further develop of EBFM. As there is a need for multispecies fishing mortalities as part of EBFM modelling, it is also suggested the multispecies proportional method of the catchable species fished in pelagic trophic levels be further investigated.

## Conclusions

This investigation shows how natural fish community ecosystems function according to marine ecology theory of biological production transfers between trophic levels. This way, ecosystem-based fisheries could mimic natural fish community processes and help define fishing mortalities for EBFM. The findings explain how modelling for EBFM could include maintenance of predator and prey biological production, fishery biomass and ecosystem stability. It is hoped that these insights for the ecological basis for ecosystem-based fishing mortalities will be of assistance to fish biologists and modellers for fishery management and its integration with the related ecological conditions that support the fisheries.
